# Reactive oxygen species–post translational modifications–central carbon metabolism regulatory loop: coordination of redox homeostasis and carbon flux allocation in plants under abiotic stress

**DOI:** 10.3389/fpls.2025.1637328

**Published:** 2025-09-29

**Authors:** Linfeng Bao, Wenya Wang, Mengyang Li, Jie Liu, Jiahao Liu, Gulizhare Alifu, Desheng Wang, Xueqi Liang, Tingyong Mao, Yunlong Zhai

**Affiliations:** ^1^ College of Agriculture, Tarim University, Alar, China; ^2^ Key Laboratory of Tarim Oasis Agriculture (Tarim University), Ministry of Education, Alar, China

**Keywords:** abiotic stress, TCA cycle, glycolysis, oxidative pentose pathway, central carbon metabolism, reactive oxygen species homeostasis, post-translational modifications

## Abstract

Reactive oxygen species (ROS) play dual roles in plants as signaling molecules and cytotoxic agents, making ROS homeostasis critical for abiotic stress adaptation. Numerous studies have shown that central carbon metabolism (CCM) provides the energy required for plant growth and maintains ROS homeostasis by coordinating energy distribution and reconfiguring metabolic streams under abiotic stress, providing energy and metabolites for plants to resist adverse conditions. As a crucial mechanism by which cells respond to short-term stress, post-translational modifications (PTMs) influence CCM by targeting and modifying its enzymes. This enables both energy and metabolic flow redistribution, enabling plants to balance growth and defense under stress conditions. In this review, we discuss the ROS–PTM–CCM interaction and how it improves plant adaptation to abiotic stress. We propose that ROS coordinate ROS homeostasis by mediating the feedback regulation of CCM through PTMs under abiotic stress. This review provides a theoretical basis for improving crop stress tolerance through PTM-targeted metabolic engineering.

## 1 Introduction

Plants, as primary producers of the Earth’s ecosystem, fix solar energy through photosynthesis and drive the carbon cycle to generate organic material and energy. However, the sessile nature of growth keeps plants exposed to dynamically changing environments. Under such dynamic environmental stresses, plants face the compounded effects of multiple abiotic stressors including drought, extreme temperatures, essential element deficiencies, and toxic elements, ultimately leading to significant crop yield losses (Gupta et al., 2020). Global warming has led to frequent extreme weather events, altering suitable production areas and increasing abiotic stress (Calvin et al., 2023). Under the dual pressure of population growth and climate change, food security is facing severe challenges (FAO, 2024). Researchers estimate that current climate trends will reduce current global yields of wheat, corn, and barley by 10%, 4%, and 13%, respectively (Lobell and Di Tommaso, 2025).

Carbon, as the core material basis of plant life activities, is the key to building organisms, driving metabolic pathways, yield formation, and environmental adaptation. Plant-immobilized CO_2_ is converted into organic carbon compounds and further distributed to growth, defense, and storage through central carbon metabolism (CCM), consisting of the glycolysis (EMP) pathway, the tricarboxylic acid (TCA) cycle, and the pentose phosphate (PPP) pathway. During seed germination, the starch or fat in the seed is broken down into glucose, which enters the EMP pathway and the TCA cycle, providing ATP and carbon skeletons (Zuo et al., 2022). During further growth, CCM connects photosynthesis and respiration, distributing the carbon fixed by photosynthesis for plant growth through the EMP pathway and the TCA cycle, and the PPP pathway provides the pentose and nicotinamide adenine dinucleotide phosphate (NADPH) required for nucleotide synthesis, promoting cell division (Huang et al., 2003). CCM provides energy for plant growth and provides energy and various metabolites for plants to resist adverse conditions by coordinating energy distribution and reconfiguring metabolic flow (Igamberdiev and Eprintsev, 2016; Liu et al., 2020; MacLean et al., 2023; Zhao et al., 2020).

Plant photosynthetic organ function and carbon metabolism balance are disturbed by abiotic stress, significantly reducing crop yield (Du et al., 2020; Kopecká et al., 2023; Muhammad et al., 2021). Reactive oxygen species (ROS) produced under stress mainly include superoxide anions (O_2_^•-^), hydrogen peroxide (H_2_O_2_), hydroxyl radicals (^•^OH), and singlet oxygen (^1^O_2_) (Mittler, 2017). ROS have dual biological characteristics in plant cells, and at a physiological concentration, they are involved in the regulation of seed germination, stomatal motility, cell differentiation, and plant adaptation to abiotic stress as key signaling molecules (Fedoreyeva, 2024; Messens, 2018; Mignolet-Spruyt et al., 2016; Vaahtera et al., 2014). However, when plants experience extreme changes in the external environment, excessive ROS accumulation can be highly cytotoxic, which makes maintaining ROS homeostasis crucial for plants to resist abiotic stress (Messens, 2018; Mignolet-Spruyt et al., 2016; Mittler et al., 2004; Vaahtera et al., 2014). Under ever-changing external environments, plants must respond quickly and adapt to environmental changes. This is influenced by multiple factors, including changes in metabolites, enzyme expression, and post-translational modifications (PTMs). Compared to the time-consuming transcription–translation of plants to regulate expression, PTMs are an important mechanism for cells to respond to short-term stress (Merchante et al., 2017; Lin and Caroll, 2018).

To respond to various stressors, PTMs link ROS with CCM by regulating protein functions (Matamoros and Becana, 2021; Wang et al., 2024; Yin et al., 2019). PTMs, such as phosphorylation, acetylation, ubiquitination, S-nitrosylation, and oxidative modification, instantly respond to fluctuations in ROS and adjust metabolic enzyme activity or localization, thereby achieving dynamic adjustment of metabolic pathways to adapt to environmental changes (Cui et al., 2024; Hashiguchi and Komatsu, 2016; Lee et al., 2020; Wang et al., 2024; Yun et al., 2011; Zhang and Zeng, 2020). We first describe the importance of CCM in maintaining ROS homeostasis under abiotic stress and then discuss how ROS mediates PTMs. We mainly focus on how ROS-mediated PTMs drive the remodeling of CCM under abiotic stress and how plants balance the energy supply with ROS scavenging capacity to adapt to stress.

## 2 Interplay mechanisms and dynamic homeostasis between CCM and ROS

### 2.1 CCM-mediated ROS production under abiotic stress

CCM and ROS generation are directly linked. The TCA cycle, as a central hub of CCM, supplies reducing equivalents (NADH and FADH_2_) to the mitochondrial electron transport chain (ETC). The ETC is a major source of intracellular ROS, with the ubiquinone oxidoreductase (Complex I) and cytochrome c reductase (Complex III) complexes being the primary sites of ROS production (Dourmap et al., 2020; Gakière et al., 2018). The concentration of TCA cycle intermediates directly regulates NADH and FADH_2_ production rates, consequently governing the electron flux into the ETC (Mailloux et al., 2007).

**Figure 1:**
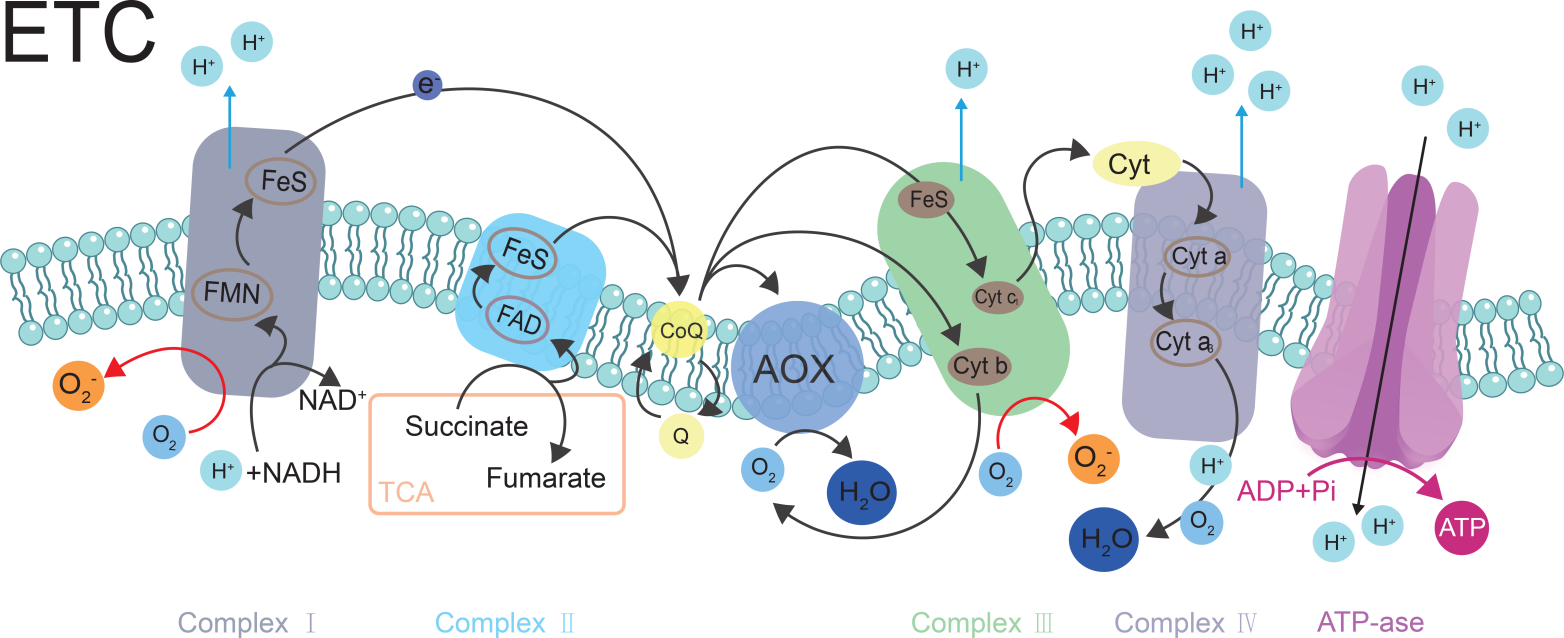
Reactive oxygen species (ROS) generation along the RETC pathway. O_2_^•−^ is formed upon the single-electron reduction of O_2_. Complexes I and III in the RETC are the major sites of O_2_^•−^ production. AOX in RECT. Black arrows indicate the electron pathway or ROS transfer, and red arrows represent O_2_^•−^ generation.

Succinate dehydrogenase (SDH, mitochondrial complex II) links the TCA cycle to the ETC and participates in mitochondrial ROS production (Jardim-Messeder et al., 2015) (**Figure 1**). When the rate of NADH production by the TCA cycle exceeds its consumption by the ETC, the ETC becomes highly reduced. This increases the probability of electron leakage from Complex I and Complex III to O_2_, thereby forming O_2_•^−^. Under stress conditions, TCA cycle disruption or metabolic reprogramming leads to succinate accumulation (Che-Othman et al., 2020). For example, elevated concentrations of succinate drive a substantial electron flux into the ubiquinone pool via Complex II. Electrons then flow in reverse from the highly reduced ubiquinol (QH_2_) back to Complex I, causing the FMN cofactor of Complex I to become highly reduced. In this highly reduced state, FMN readily leaks electrons to O_2_, generating substantial O_2_•^−^ (**Figure 1**). The TCA cycle extends beyond merely supplying reducing equivalents to the ETC. Through dynamically regulating the concentrations and flux of its intermediates, the TCA cycle directly influences the redox status and stability of the mitochondrial ETC, thereby serving as a central metabolic hub for both physiological ROS signaling and stress-induced ROS bursts.

However, CCM-mediated ROS production is not confined to mitochondria. Under abiotic stress, photorespiration acts as a pivotal component of CCM remodeling and becomes a significant source of ROS through the reaction catalyzed by glycolate oxidase (GOX) in peroxisomes, using O_2_ as an electron acceptor and directly producing H_2_O_2_ (Hu et al., 2023; Wang et al., 2022; Williams et al., 2018). Photorespiration originates from the oxygenase activity of the core CCM enzyme Rubisco, which converts the Calvin cycle intermediate RuBP into phosphoglycolate, subsequently generating glycolate, which marks a shift of carbon flux from the carboxylation pathway to the photorespiratory pathway (Busch, 2020; Fernie and Bauwe, 2020; Niessen et al., 2007; Timm et al., 2024). Notably, factors regulating the photorespiratory flux and its substrate (glycolate) levels intrinsically belong to the CCM network and affect ROS production.

Rubisco plays a dual core role: it is not only the initiator of photorespiration (via its oxygenase activity) but also serves as a pivotal hub for carbon loss and redox regulation throughout the entire photorespiration pathway (Prywes et al., 2023). Rubisco has a much lower affinity for CO_2_ (higher Km) than O_2_. This means that at equal concentrations, Rubisco binds O_2_ more readily, thus catalyzing the oxygenation reaction. Thus, plants evolved the carbon concentrating mechanism as an adaptive strategy to increase the CO_2_ concentration at the Rubisco carboxylation site. Carbon anhydrases (CAs) and CO_2_/HCO_3_^−^ transporters maintain a high stromal CO_2_/O_2_ ratio, thereby suppressing Rubisco oxygenase activity, reducing phosphoglycolate/glycolate production, and decreasing ROS generation (He et al., 2023; Ślesak et al., 2017; Wang et al., 2022).

Intermediate photorespiratory transporters directly affect redox homeostasis (Corpas et al., 2019a; Corpas et al., 2019b; Sánchez-McSweeney et al., 2021; Wang et al., 2022). Previous research showed that loss of function in the chloroplast membrane K^+^/H^+^ antiporters AtKEA1 and AtKEA2 affected GOX and redox homeostasis, while the *kea1kea2* mutant exhibited enhanced drought tolerance (Sánchez-McSweeney et al., 2021). In another study, increased H_2_O_2_ in the *Arabidopsis* catalase-deficient mutant *cat2* triggered SA-dependent cell death, while reducing GOX activity alleviated this effect (Wang et al., 2022).

Therefore, similar to the way TCA cycle intermediate accumulation (e.g., succinate) triggers ROS through affecting ETC reduction status in mitochondria, in the chloroplast/photorespiratory pathway, the stress-induced elevation of photorespiratory flux and accumulation of the key substrate glycolate are the core mechanisms driving ROS production.

### 2.2 CCM dynamically regulates ROS homeostasis under abiotic stress

Under abiotic stress, the transport and distribution mechanisms of carbon sources are at the core of plant stress resistance and yield formation. Abiotic stress affects the formation of carbon sources by destroying chloroplasts, reducing the photosynthetic pigment content, blocking ETCs, and reducing the carbon fixation efficiency (Bose et al., 2017; Chen et al., 2020; Nath et al., 2013; Qiao et al., 2024; Yoshioka et al., 2006). In the case of blocked carbon sources, CCM balances plant defense and growth in the face of adversity by maintaining energy homeostasis and redistributing metabolites.

**Figure 2:**
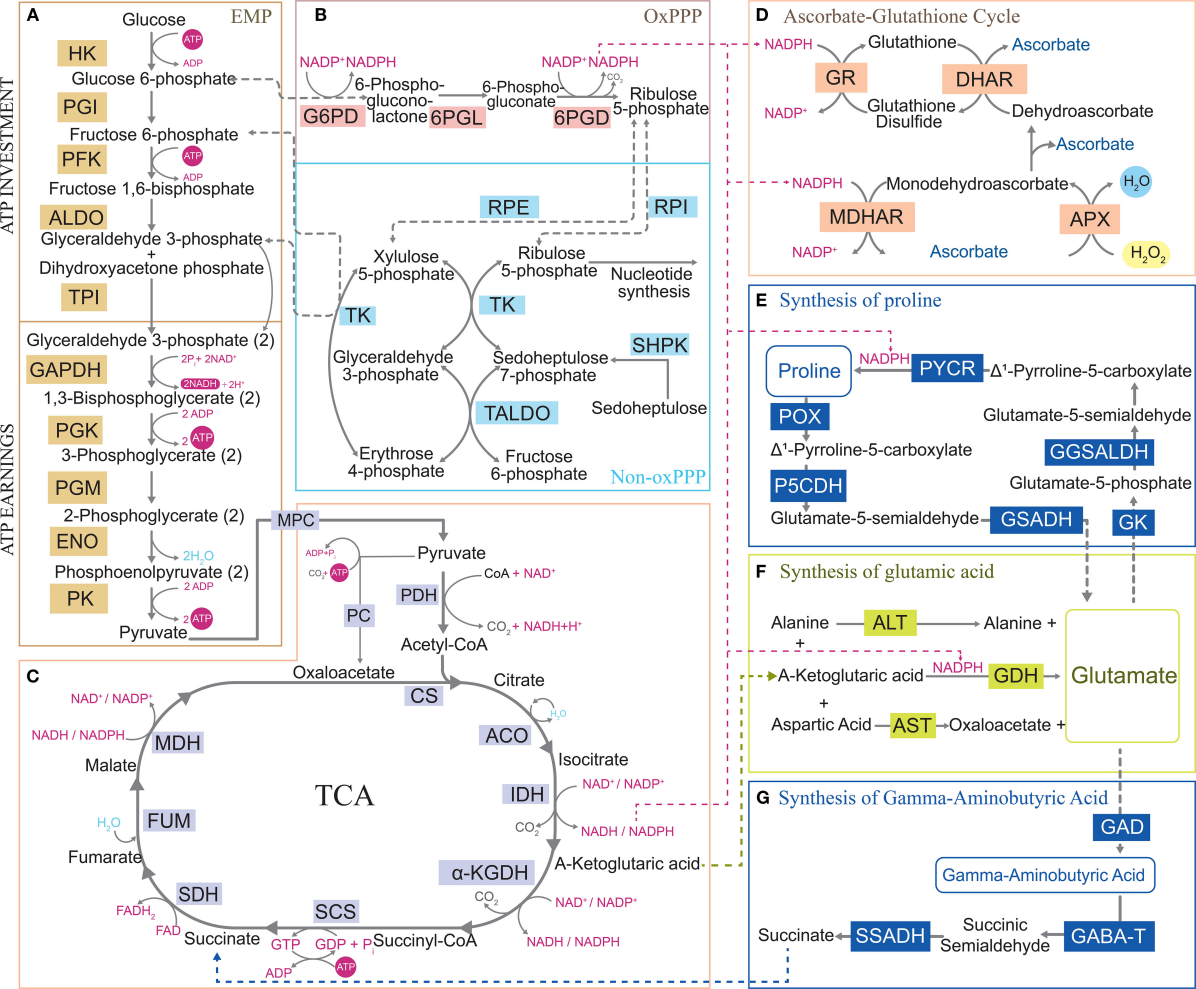
Central carbon metabolism maintains energy homeostasis and mediates metabolite remodeling under abiotic stress. The NADPH produced in central carbon metabolism supplies the ascorbic acid glutathione cycle to eliminate reactive oxygen species and provides reducing power for the synthesis pathways of other substances. Communication with other metabolic pathways demonstrates the flexibility of central carbon metabolism under abiotic stress. **(A)** Glycolysis pathway. **(B)** Pentose phosphate pathway. **(C)** Tricarboxylic acid cycle. **(D)** Ascorbic acid glutathione cycle. **(E)** Proline synthesis pathway. **(F)** Glutamic acid synthesis pathway. **(G)** gamma-aminobutyric acid synthesis pathway. HK, Hexokinase; PGI, Phosphoglucose isomerase; PFK, Phospho-fructokinase-1; ALDO, Aldolase; TPI, Triose phosphate isomerase; GAPDH, Glyceraldehyde 3-P dehydrogenase; PGK, Phosphoglycerate kinase; PGM, Phosphoglycerate mutase; ENO, Enolase; PK, Pyruvate kinase; G6PD, Glucose-6-phosphate dehydrogenase; 6PGL, 6-Phosphoglucono lactonase; 6PGD, 6-Phosphogluconate dehydrogenase; RPE, Ribulose-phosphate 3-epimerase; RPI, Ribose-5-phosphate isomerase; TK, Transketolase; SHPK, Sedoheptulokinase; TALDO, Transaldolase; MPC, Pyruvate carrier; PC, Phosphoenolpyruvate carboxykinase; PDH, Pyruvate dehydrogenase complex; CS, Citrate synthase; ACO, Aconitate hydratase; IDH, Isocitrate dehydrogenase; α-KGDH, α-Ketoglutarate dehydrogenase complex; SCS, Succinyl-CoA synthetase; SDH, Succinate dehydrogenase; FUM, Fumarate hydratase; MDH, Malate dehydrogenase; GR, Glutathione reductase; DHAR, Dehydroascorbate reductase; MDHAR, Monodehydroascorbate reductase; APX, Ascorbate peroxidase; PYCR, Pyrroline-5-carboxylate reductase; GGSALDH, Glutamate-γ-semialdehyde dehydrogenase; GK, Glutamate kinase; GSADH, Glutamate-5-semialdehyde dehydrogenase; P5CDH, Δ^1^-Pyrroline-5-carboxylate dehydrogenase; POX, Proline oxidase; ALT, Alanine transaminase; GDH, Glutamate dehydrogenase; AST, Aspartate transaminase; GAD, Glutamate decarboxylase; GABA-T, Gamma-aminobutyric acid transaminase; SSADH, Succinic semialdehyde dehydrogenase; ROS, Reactive oxygen species; H_2_O_2_, Hydrogen peroxide; ATP, Adenosine triphosphate; ADP, Adenosine diphosphate; GTP, Guanosine triphosphate; NAD^+^, Nicotinamide adenine dinucleotide; NADP^+^, Triphosphopyridine nucleotide; NADH, Nicotinamide adenine dinucleotide; NADPH, Triphosphopyridine nucleotide.

The change in the ATP/ADP ratio directly affects the energy state of cells, while the change in the NADPH/NADP+ ratio reflects the reduction state of cells (Chandel, 2021; Hu et al., 2020). Under abiotic stress, the ATP/ADP and NADPH/NADP+ ratios decrease. Plants sense these ratios, reprogram CCM, and redistribute metabolites to preferentially meet ATP and NADPH demands under stress (Hu et al., 2020). Under abiotic stress, plants upregulate the energy supply by activating key enzymes in the EMP pathway. This enhances rapid glucose breakdown to ATP and bypasses ATP-requiring steps (e.g., via metabolic flux redistribution), thereby minimizing ATP consumption and increasing the ATP/ADP ratio, which is a critical strategy for stress adaptation (Dobrota, 2006; Rizhsky et al., 2002). During oxidative stress, the decreased NADPH/NADP+ ratio triggers CCM to dynamically adjust fluxes through the PPP pathway and TCA cycle, replenishing the intracellular NADPH pool (Moon et al., 2020; Stincone et al., 2015). As a NADPH-driven H_2_O_2_ scavenging pathway, the ascorbate-glutathione (AsA-GSH) cycle mitigates ROS accumulation by upregulating enzyme activity, overexpressing enzymes in the pathway, and increasing AsA and GSH levels, which enhances plants’ tolerance to abiotic stress (Hasanuzzaman et al., 2019) (**Figure 2D**). This suggests that CCM improves the ability of plants to maintain redox homeostasis by providing ATP and NADPH to the AsA-GSH cycle. A study has shown that wheat activates the PPP pathway and optimizes energy metabolism to boost NADPH and ATP production, which sustains cellular redox and energy homeostasis, improving drought stress adaptation (Chen et al., 2004). As a key link in NADPH production in the PPP pathway, the glucose-6-phosphate (G6P) shunt oxidizes G6P to glucose-6-phosphate lactone through glucose-6-phosphate dehydrogenase (G6PDH), which accompanies NADPH production (**Figure 2B**). Studies have found that plants activate G6P diversion at high temperatures to reintroduce unlabeled carbon into the Calvin–Benson cycle. It not only generates NADPH but also maintains the flexibility of carbon metabolism under high-temperature stress and provides a metabolic regulation approach for plants to cope with environmental changes (Sharkey et al., 2020).

Metabolite accumulation can prevent adverse toxic effects. Certain metabolites, such as organic acids, proline (Pro), betaine, and trehalose, significantly accumulate, playing a role in osmotic protection, maintaining cellular homeostasis, and indirectly reducing ROS generation (Krasavina et al., 2014). Plants alter metabolite allocation by reprogramming CCM, directly or indirectly providing key metabolites required to adapt to stress conditions. Abiotic stress triggers changes in enzyme activity during the TCA cycle, resulting in fluctuations in specific organic acid levels (Das et al., 2019). Malate dehydrogenase (MDH) catalyzes the reversible conversion of oxaloacetic acid to malic acid and plays an important regulatory role in plants’ resistance to metal toxicity (Liu et al., 2023; Zhou et al., 2018). *GmME1* is a cytoplasmic malate enzyme that increases malic acid and citric acid synthesis and balances the aluminum-induced efflux of malic acid and citric acid, increasing tolerance to aluminum stress (Zhou et al., 2018). The increase in α-ketoglutarate (α-KG) under stress conditions is caused by isocitrate dehydrogenase overexpression and the decrease in α-ketoglutarate dehydrogenase (KGDH) activity under stress conditions (Legendre et al., 2020; Lemire et al., 2010; Mailloux et al., 2009). KGDH is inhibited in the TCA cycle, causing α-KG to enter other metabolic pathways. The gamma-aminobutyric acid (GABA) shunt starts with α-KG in the TCA cycle, which is converted into glutamic acid (Glu) by glutamic acid dehydrogenase (GDH) and then into GABA by glutamic acid decarboxylase (Benidickson et al., 2023). GABA shunt is crucial to plant stress resistance. In guard cells, GABA modulates anion channels to regulate stomatal aperture, reducing transpirational water loss and enhancing plant water use efficiency and drought tolerance (Xu et al., 2021). However, under salt stress, GAD activity is enhanced, promoting the conversion of Glu to GABA. GABA is subsequently metabolized to succinate via GABA transaminase (producing succinic semialdehyde as an intermediate) and succinate semialdehyde dehydrogenase, bypassing the inhibited mitochondrial α-ketoglutarate dehydrogenase complex. This metabolic rerouting reintegrates carbon sources into the TCA cycle. Concurrently, the abundance of α-ketoglutarate (α-KG)/malate transporters increases, facilitating α-KG efflux for amino acid synthesis and coordinating the balance between carbon and nitrogen metabolism (Che-Othman et al., 2020) (**Figure 2G**). A recent study has shown that alkaline stress upregulates *MdSINA2* expression, which triggers ubiquitin-mediated degradation of *MdNAC104*, thereby relieving its inhibition of *MdGAD1/3*; this cascade promotes the conversion of Glu to GABA and enhances GABA synthesis (Che-Othman et al., 2020). Increased GABA synthesis acts not only as a stress-induced signaling molecule but also mitigates the stress-triggered energy imbalance by replenishing succinate, a TCA cycle intermediate. It also enhances stress tolerance through membrane potential regulation, ion toxicity reduction, and antioxidant capacity augmentation. Similarly, Glu serves as the primary precursor for Pro synthesis, which depends on α-KG through the catalytic reaction of GDH (Givan et al., 1970) (**Figure 2E**). Under drought stress, the expression of TCA cycle-related proteins, such as citrate synthase, pyruvate dehydrogenase E1α subunit (PDHE1α-2), and aconitase, is upregulated in wheat, providing energy and metabolic intermediates for Pro synthesis. The upregulation of sucrose synthase and raffinose synthase and the downregulation of sucrose-degrading enzymes promotes sucrose accumulation. Sucrose synergistically acts as an osmotic protectant with Pro, and the EMP pathway supplies carbon sources for Pro synthesis (Wang et al., 2019). Under salt stress, *atpao5* mutants accumulate higher levels of Pro, TCA cycle intermediates, and sugars, enhancing salt stress tolerance. These findings demonstrate that Pro synthesis may be enhanced through the metabolic flux of the TCA cycle, providing a precursor (e.g., α-KG) for its biosynthesis (Zarza et al., 2017).

These studies highlight the communication between CCM and other metabolic branches under abiotic stress. Although the underlying mechanisms remain elusive, these findings demonstrate that plants dynamically coordinate energy allocation and metabolite remodeling via CCM to maintain interactions with diverse metabolic pathways. This coordination enables plants to fine-tune their metabolic states and preserve ROS homeostasis, thereby acclimating them to environmental fluctuations. To unravel how CCM is reprogrammed during abiotic stress, deciphering the roles of ROS at distinct concentration levels or spatiotemporal dynamics in driving CCM remodeling is a critical focus.

### 2.3 ROS-dependent regulation of CCM enzymes

Under stress, excess ROS harms plants. Before CCM maintains energy homeostasis and balances metabolic flux, low-level ROS (nM to μM H_2_O_2_) act as signaling molecules during early stress. These ROS sense stress, trigger signaling cascades (Ca^2+^ and MAPK), and regulate key metabolic enzymes. This regulation involves the reversible oxidation of cysteine residues (Li and Kim, 2022; Mohanta et al., 2018; Mittler et al., 2022; Vogelsang and Dietz, 2022; Zhou et al., 2022). During early iron deficiency, trace H_2_O_2_ acts as a signaling molecule and triggers the MAPK signaling cascade, activating the kinase *MxMPK6-2*, which phosphorylates the transcription factor *MxbHLH104*. This phosphorylation enhances MxbHLH104 activity, thereby increasing iron uptake efficiency under iron-deficient conditions. Therefore, *MxMPK6-2* overexpression confers greater tolerance to iron deficiency through responding to ROS signals and phosphorylating/activating MxbHLH104 (Li et al., 2021). Following H_2_O_2_ accumulation in the apoplast, the rice aquaporin (AQP) OsPIP2;2 is activated through the phosphorylation of its serine residue S125. Activated *OsPIP2;2* then transports H_2_O_2_ into the cytosol, where cytosolic H_2_O_2_ activates the MAPK cascade MPK3/6. MPK3/6 activation initiates defense gene expression. Concurrently, cytosolic H_2_O_2_ drives the transcription factor *OsmaMYB* to translocate to the nucleus, where it regulates defense gene expression. These two pathways act together to enhance plant resistance (Zhang et al., 2022).

Research has shown that 5-aminolevulinic acid (ALA) pretreatment enhances cold-triggered oxidative stress tolerance in tomato through inducing H_2_O_2_ signaling and subsequent crosstalk with redox signaling (Liu et al., 2019). In another study, exogenous H_2_O_2_ priming significantly improved the growth and antioxidant defense capacity of two plant species under salt stress, drought stress, and their combination (Ellouzi et al., 2017). Similarly, H_2_O_2_ priming likely enables plants to “remember” and “decode” early H_2_O_2_ signals through its signaling function, facilitating the more effective activation of plant defense responses when facing stress in the future. These findings indicate the role of early ROS signals in signal transduction.

During the initial phase of stress, ROS modifies key CCM enzymes through reversibly oxidizing cysteine residues at their active sites or regulatory sites, inducing S-sulfenylation, the formation of disulfide bonds, or S-glutathionylation (Dalle-Donne et al., 2007; Vogelsang and Dietz, 2022). This inhibition reduces or inactivates the target enzyme, thus rapidly and reversibly altering CCM. The key physiological purpose of this process is to balance energy and reduce power supply and demand; redirect resources to defense systems; and allow rapid recovery after exposure to stress. Thioredoxin and glutathione reductase systems inside the cell reverse these oxidative modifications, making the process an adaptive mechanism (Holmgren, 1985). The interaction among ROS, PTMs, and CCM serves as one of the core mechanisms in plant defense against abiotic stress. The following section details the molecular mechanisms by which ROS regulate CCM through mediating PTMs.

## 3 ROS-mediated PTMs drive the reconfiguration of the CCM network

PTMs dynamically regulate protein activity by covalently attaching or detaching specific chemical groups (e.g., phosphate and acetyl) or altering protein conformations. Key PTMs, including phosphorylation, acetylation, ubiquitination, glycosylation, and S-nitrosylation, act as molecular switches that fine tune protein structures and functions through site-specific chemical modifications (Baoxiang et al., 2023; Kumar et al., 2021; Paudel et al., 2016; Sharma et al., 2021; Shen et al., 2020; Yun et al., 2011). Further, PTMs interact by enhancing or inhibiting each other’s functions and integrating dispersed PTMs into a coordinated PTM network, which mediates adaptive protein modifications to counteract stress. As previously mentioned, ROS exhibit a dual biological nature. Plants maintain ROS homeostasis by tightly regulating their production and scavenging. In homeostasis, ROS act as signaling molecules to activate downstream pathways that regulate growth and development and rapidly respond to abiotic stress, playing a pivotal role in balancing cellular homeostasis and stress adaptation. Considering dynamic redox changes in plant cells, ROS induce diverse PTMs by specifically modifying the key amino acid residues on target proteins. ROS-triggered PTMs exhibit concentration-dependent effects, highlighting the regulatory interplay between ROS levels and PTMs in stress responses.

### 3.1 Specificity of ROS signaling

**Figure 3:**
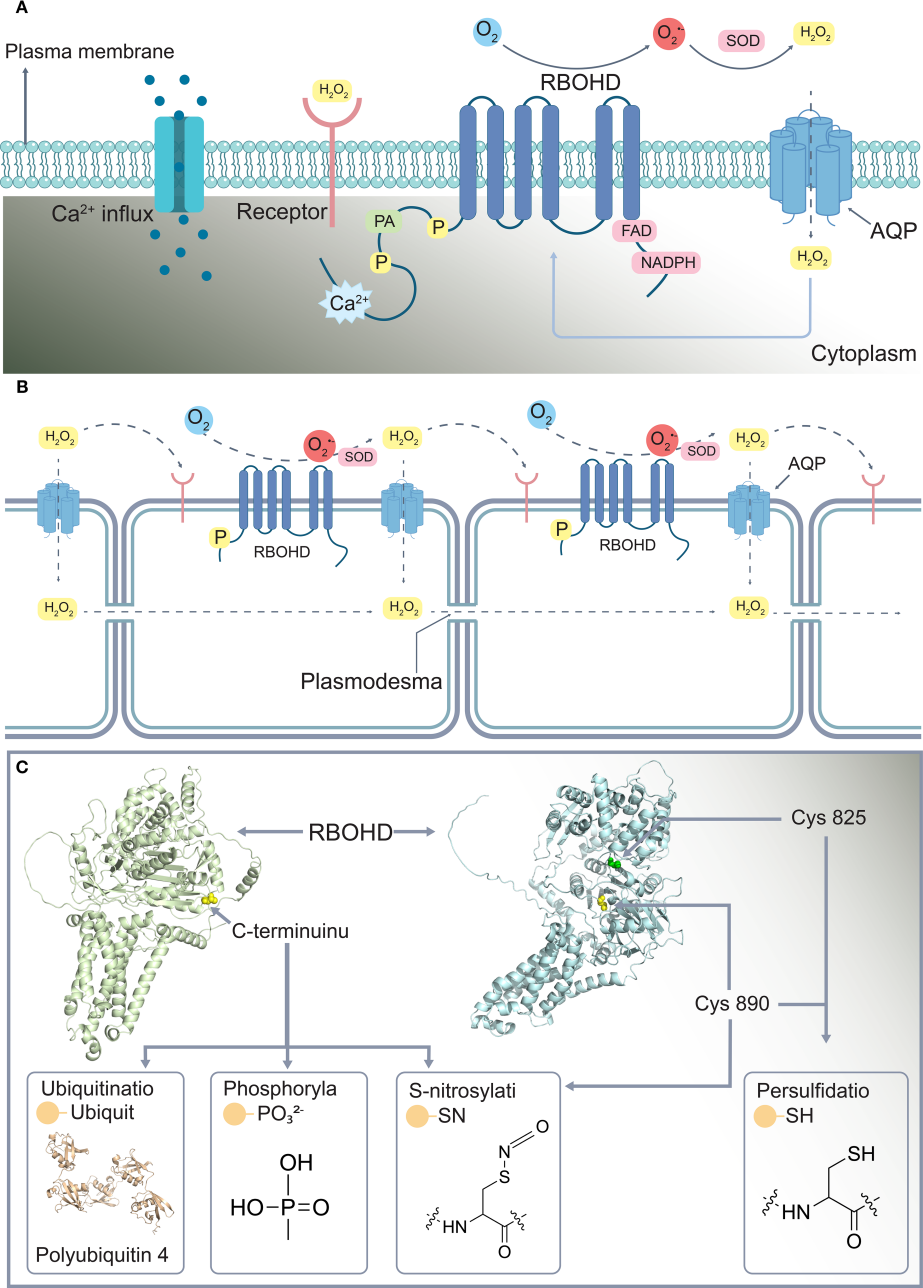
Specificity of reactive oxygen signaling. **(A)** ROS signals communicate through RBOHD, and other signaling pathways are transmitted in cells. **(B)** ROS signals communicate with other signaling pathways through RBOHD to spread between cells. **(C)** Post-translational modification sites on RBOHD. RBOHD, Respiratory burst oxidase homolog protein D; H_2_O_2_, Hydrogen peroxide; SOD, Superoxide dismutase; PA, Phosphatidic acid; AQP, Aquaporin. In **Figure 3**, *Arabidopsis* RBOHD uses the AF–Q9FIJ0–F1–v4 AlphaFold prediction model as a reference (https://alphafold.ebi.ac.uk/entry/Q9FIJ0), while *Arabidopsis thaliana* Polyubiquitin 4 uses AlphaFold prediction model AF–P0CH32–F1–v4 (https://alphafold.ebi.ac.uk/entry/P0CH32); for example, modify the site with a PyMOL software tag.

In plants, compartments are spatially organized at multiple hierarchical levels. At the systemic level, they are distributed across organs and tissues, and within a single tissue or organ, distinct compartments exist among cell types. At the subcellular level, organelles (e.g., chloroplasts, mitochondria, and peroxisomes) and the cytoplasm are partitioned into specialized reaction zones. Unlike most signaling molecules, ROS are independently generated in nearly all cellular compartments, which enables plants to compartmentalize redox reactions, thereby mitigating the risks of redox imbalance and allowing dynamic coordination of stress responses and metabolic adaptations (Bar-Peled and Kory, 2022; Mittler et al., 2022). ROS in different compartments link Ca^2+^, phosphorylation, phosphatidic acid, and redox signals through the functional integration of respiratory burst oxidase homolog D (RBOHD), AQPs, Ca^2+^ channels, and multiple enzymes, driving ROS propagation within and between compartments (Bartoli et al., 2013; Feng et al., 2024; Rodrigues et al., 2017; Zandalinas et al., 2020) (**Figures 3A, B**). In addition to being generated in different cellular compartments, ROS can be transported to other cells and tissues through the opening and closing of AQPs between compartments, which is regulated by PTMs, and are connected to different signaling pathways (Bienert et al., 2007; Moon et al., 2020; Qing et al., 2016; Qiu et al., 2020; Rodrigues et al., 2017). Therefore, ROS heterogeneity arises from variations in species, such as H_2_O_2_ and O_2_^•-^, and is closely linked to their dynamic production and transport within cellular compartments or tissues. ROS directly regulate metabolic enzyme activity at production sites and are transported between cells via transmembrane mechanisms, remotely affecting CCM balance in distal tissues. This establishes a local–systemic signaling network in plants (Bao et al., 2024; Mittler et al., 2022).

The redox signals caused by ROS are mainly produced by mediating the corresponding oxidative post-translational modifications (OxiPTMs) of proteins and have been associated with other signaling pathways (Mittler et al., 2022). Under stress, Cys308 of tryptophan synthase β subunit1 is sulfinylated by H_2_O_2_, decreasing tryptophan (Trp) and auxin contents and inhibiting plant growth (Liu et al., 2022b). In addition to directly mediating modifications, H_2_O_2_-induced activation of Ca^2+^ channels is indirectly achieved through covalent modification of two pairs of cysteine (Cys) residues in the extracellular domain of its activated receptor kinase HPCA_1_ (hydrogen peroxide-induced Ca^2+^ increases), leading to HPCA_1_ autophosphorylation (Wu et al., 2020). However, in the process of mediating PTMs, the enzyme responsible for ROS production is regulated by PTMs during ROS signaling. Among these enzymes, RBOHD, which is involved in ROS generation and long-distance signaling, undergoes C-terminal modifications, including phosphorylation, ubiquitination, and S-nitrosylation (Lee et al., 2020; Miller et al., 2009; Yun et al., 2011). At low concentrations, S-nitrosothiols positively regulate the activity of enzymes, such as *AtRBOHD*, which promotes ROS production, amplifying signaling. In contrast, high-concentration S-nitrosylation at *AtRBOHD* (Cys890) suppresses ROS generation (Yun et al., 2011). However, the persulfidation of RBOHD at Cys825 and Cys890 promotes abscisic acid (ABA)-dependent stomatal closure and enhances ROS production (Shen et al., 2020) (**Figure 3C**).

In addition to the toxic effects caused by excessive ROS production, plants exploit the specificity of ROS as signaling molecules to activate downstream transduction pathways and coordinate broader signaling networks by mediating epigenetic modifications. Not only do plants utilize the distinct properties or ROS to maintain redox homeostasis, but they also develop unique metabolic regulatory processes. ROS-triggered PTMs either directly or indirectly act on plant metabolic networks. Such metabolic reprogramming, driven by redox signaling, demonstrates the involvement of ROS in the functional execution of proteins.

### 3.2 Molecular mechanisms and functions of ROS-dependent PTMs

In plants, OxiPTM, which is specifically induced under oxidative stress by ROS, reactive nitrogen species (RNS), and reactive sulfur species (RSS), modifies specific amino acid residues of proteins, mediating signaling, stress responses, and metabolic regulation (Zhou et al., 2023). Here, we focus on oxidative reactions mediated by ROS.

#### 3.2.1 PTMs directly mediated by ROS

**Figure 4:**
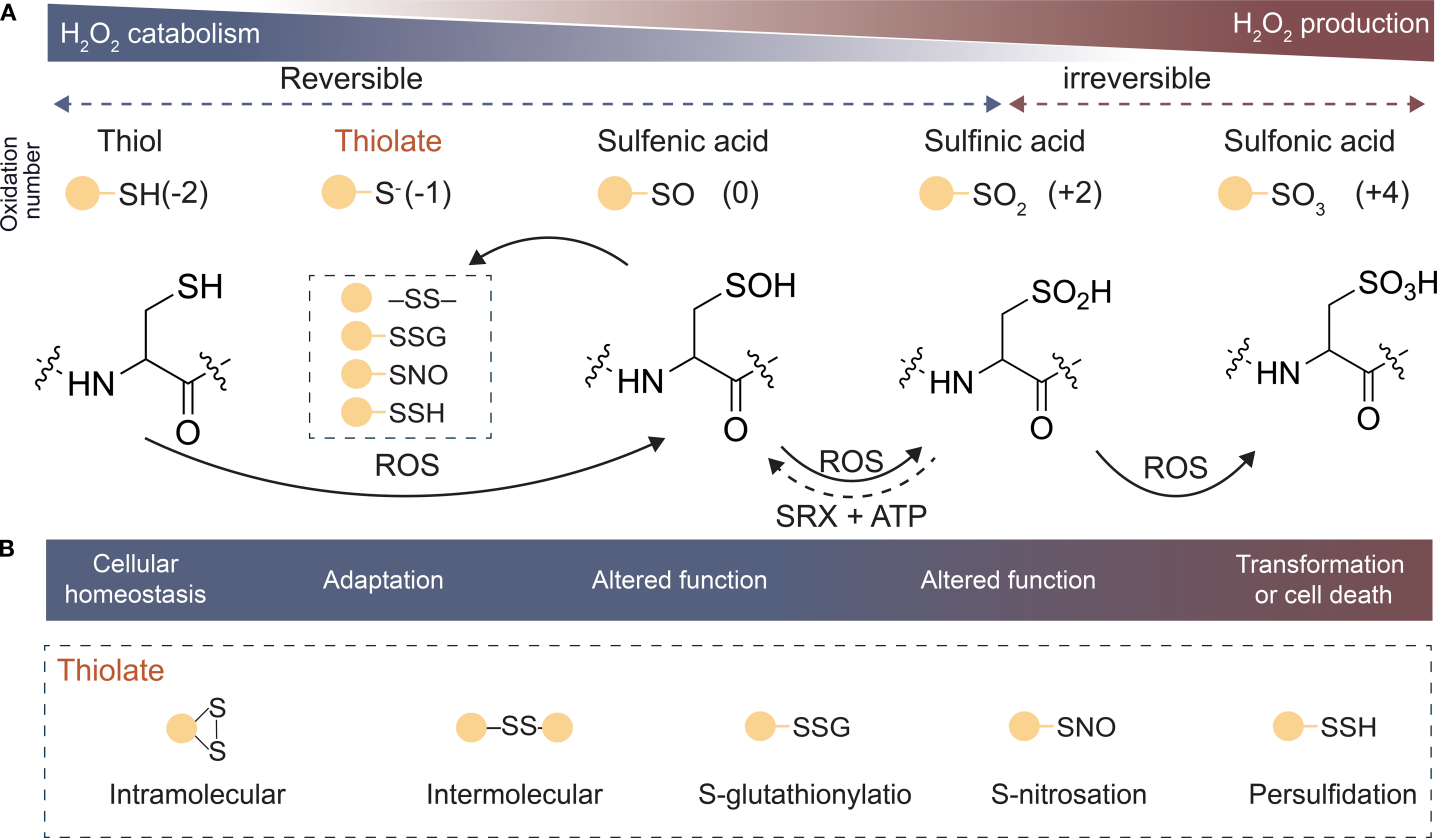
Oxidation states of different sulfur (S) atoms in cysteine. **(A)** Oxidation state of S in proteins as H_2_O_2_ is produced, ranging from mercaptans (−2) to sulfonic acids (+4). **(B)** Main reversible oxidation type of mercaptans, intramolecular disulfide, intermolecular disulfide, S-glutathionylation, S-nitrosation, and Persulfidation. Abbreviations: ATP, Adenosine triphosphate; SRX, Sulfiredoxin; H_2_O_2_, Hydrogen peroxide; ROS, Reactive oxygen species.

The PTMs induced by ROS and its reversibility are key features of ROS signal transduction (Miao et al., 2006). Among ROS, H_2_O_2_, as a moderate oxidant, effectively oxidizes diverse proteins to form distinct *OxiPTMs*. This selective oxidation likely arises from factors such as the charge distribution of amino acids adjacent to thiol groups, determining the specific modification patterns of different proteins. As the predominant *OxiPTM*, ROS-mediated redox modifications of protein Cys residues serve as the primary pathway for functional regulation. Cys is highly susceptible to redox reactions due to the sulfur atom in its side chain, which exhibits multiple oxidation states. The multivalent nature of sulfur not only confers chemical versatility but also enables dynamic functional regulation (Paulsen and Carroll, 2013; Zhou et al., 2023). In biological systems, the thiol group (–SH) is the most reactive moiety in protein residues. As oxidative levels increase, the –SH on the side chain of Cys residues is initially oxidized to sulfenic acid (–SOH), which can participate in disulfide bond (–SS–) formation or glutathionylation (–SSG) or be further oxidized to sulfinic acid (–SO_2_H) and sulfonic acid (–SO_3_H) (Dalle-Donne et al., 2007; Vogelsang and Dietz, 2022; Zhou et al., 2022) (**Figure 4**). Under physiological conditions, protein sulfenylation and sulfinylation are typically irreversible modifications that lead to protein degradation. However, under specific conditions, certain proteins that undergo sulfinylation can be reductively repaired. For example, some peroxiredoxins can be reduced by sulfiredoxin (SRX) in the presence of ATP (Iglesias-Baena et al., 2011) (**Figure 4**). This indicates that the diverse and complex reductase systems in cells mediate the reduction of the aforementioned modifications, except for sulfonic acid. For instance, disulfide bonds (–SS–) and glutathionylation can be reduced by the thioredoxin/thioredoxin reductase (Trx/TrxR) and glutaredoxin/glutathione reductase systems, respectively (Holmgren, 1985).

As sensitive targets for H_2_O_2_-dependent oxidation, thiol peroxidases, such as glutathione peroxidases (GPXs) and peroxiredoxins, rely on their Cys residues for functionality. In addition to their antioxidant capacity, they also function as redox sensors to transfer H_2_O_2_ signals to diverse targets (Zhou et al., 2022). Rice GPX1 senses intracellular H_2_O_2_ and transduces oxidative signals. Under osmotic stress, cytosolic GPX1 is oxidized to form intramolecular disulfide bonds (–SS–), triggering its translocation into the nucleus. In the nucleus, GPX1 interacts with the downstream transcription factor bZIP68 (basic-region leucine-zipper 68), which induces bZIP68 oligomerization, activating the expression of stress-responsive genes (Zhou et al., 2022). Cys51 of PRXIIB in the cytoplasm of *Arabidopsis* is oxidized by H_2_O_2_, which transmits oxidative signals through the formation of an intermolecular disulfide bond (–SS–) with the Cys residue of phosphatase ABI2, inhibiting ABI2 phosphatase activity and regulating stomatal closure (Bi et al., 2022). However, AtHSFA8, a redox-sensitive transcription factor, undergoes H_2_O_2_-triggered conformational changes, which are likely mediated by the formation of an intramolecular disulfide bond (–SS–) between Cys24 and Cys269. This structural alteration facilitates the nuclear translocation of *AtHSFA8*, enabling its binding to target gene promoters (Giesguth et al., 2015). The sulfinylation of Cys308 in tryptophan synthase β subunit1 (TSB1), which is mediated by H_2_O_2_, inhibits its enzymatic activity, reducing Trp and auxin levels and thereby suppressing plant growth. This modification alleviates the inhibitory effect on BG1, activating the ABA signaling pathway to enhance stress responses. This increases ABA accumulation, further enhancing the plant’s adaptive capacity under adverse conditions (Liu et al., 2022b). During leaf senescence, the decline in chlorophyll content is accompanied by increased H_2_O_2_ accumulation and suppressed CAT-related gene expression. Compared to the wild type, the H_2_O_2_-accumulating mutant *cat2-1* exhibits accelerated senescence and reduced magnesium chelatase (CHLI_1_) activity. These findings indicate that H_2_O_2_ inhibits chlorophyll biosynthesis by sulfonylating CHLI_1_ during leaf senescence, thereby suppressing its enzymatic activity (Wang et al., 2024). However, under salt stress, H_2_O_2_ production induces the sulfinylation of Cys74 in chloroplast triose phosphate isomerase (pdTPI), leading to methylglyoxal (MG) accumulation and plant growth inhibition (Fu et al., 2023). In *Arabidopsis thaliana*, H_2_O_2_ suppresses the enzymatic activity of GSNOR_1_ in *Arabidopsis thaliana* by mediating S-sulfenylation at its Cys-284 residue during ovule development. Mutation in GSNOR_1_ disrupts nitric oxide (NO) homeostasis in the pistil, resulting in defective ovule development (Sun et al., 2025).

Notably, ROS-mediated PTMs exhibit significant temporal dynamics during stress responses. At early stress stages, PTMs induced by low ROS levels (e.g., disulfide bond formation, S-nitrosylation, and glutathionylation) are typically rapid, dynamic, and reversible, enabling rapid signal transduction and adaptive regulation during initial oxidative stress (Bi et al., 2022; Giesguth et al., 2015). These modifications primarily function in signal transmission and adaptive network reprogramming. However, as stress intensifies and prolongs, accumulated ROS overwhelm cellular redox homeostasis, driving sustained oxidative stress and the predominance of irreversible PTMs (Dalle-Donne et al., 2007; Vogelsang and Dietz, 2022; Iglesias-Baena et al., 2011). This shift from reversible regulation to irreversible protein damage and clearance ultimately triggers irreversible cellular damage or death. Thus, the specificity of ROS signaling can be seen not only in spatial compartmentalization and molecular targets but also in PTM types and their temporal dynamics.

#### 3.2.2 PTMs indirectly mediated by ROS

The above-mentioned dynamic framework is also applicable to the oxidative modification of non-cysteine. S-nitrosylated proteins are significantly enriched in multiple metabolic processes and stress responses, indicating that S-nitrosylation likely serves as a key regulatory mechanism in these pathways. As one of the most extensively studied OxiPTMs, S-nitrosylation is predominantly mediated by RNS. Although no direct role has been identified for ROS, they indirectly regulate S-nitrosylation by modulating NO production (Cui et al., 2024; Hu et al., 2015; Iglesias et al., 2018; Puyaubert et al., 2014). ROS and NO production and scavenging are interdependently regulated, and this crosstalk is closely linked to their biosynthesis in plants under abiotic stress (Corpas et al., 2019). NO triggers RBOHD-mediated ROS production, and H_2_O_2_ inhibits S-nitrosoglutathione reductase (GSNOR) activity, indicating direct crosstalk between ROS and NO signaling (Arruebarrena Di Palma et al., 2020; Kovacs et al., 2016; Rasul et al., 2012). The synergistic and antagonistic interplay between ROS and RNS is noteworthy. For example, H_2_O_2_ can enhance S-nitrosylation levels by activating nitrate reductase to promote NO synthesis (Bright et al., 2006; Corpas et al., 2022). However, under stress, mitochondrial ROS production is triggered to activate MAPK signaling. Downstream of the MAPK pathway, the Ser847 residue of 90-kDa ribosomal S6 kinase 1 (RSK1) undergoes phosphorylation. Nitric oxide synthase is phosphorylated, and both events reduce NO production (Chang et al., 2012; Takata et al., 2020). This indicates that ROS indirectly affect S-nitrosylation by influencing NO production.

This discussion primarily focuses on the redox modifications of Cys. However, redox regulation extends beyond Cys, with oxidative modifications to other amino acids being equally significant. Further, in addition to the direct effects of ROS on PTMs, their indirect regulatory mechanisms merit thorough exploration, given the profound impact of such redox events on protein structures and functions.

## 4 ROS-mediated PTM and central carbon metabolism crosstalk

### 4.1 PTM crosstalk

In plants, individual proteins (particularly enzymes) are often dynamically regulated by multiple PTMs. These modifications do not exist in isolation but form regulatory networks through complex crosstalk mechanisms. For example, under stress conditions, ROS and RNS target Cys residues in proteins are generated via reversible *OxiPTMs*, inducing S-nitrosylation, the formation of disulfide bonds, and S-glutathionylation (Vogelsang and Dietz, 2022; Zhou et al., 2022, Zhou et al., 2023; Liu et al., 2022b; Wu et al., 2020). These *OxiPTMs* further cooperate or display antagonistic interactions with PTMs through processes such as phosphorylation and ubiquitination, forming a dynamically interacting PTM regulatory network. This network ultimately precisely regulates protein activity, stability, subcellular localization, and protein–protein interactions, thereby mediating the reprogramming of plant developmental and stress response biological functions (Liu et al., 2023; Rui et al., 2024; Uhrig et al., 2019; Zhang and Zeng, 2020; Zhang et al., 2025).

In immune regulation, CPK28 functions as a negative immune regulator in *Arabidopsis thaliana*. Its functional stability is governed by a cascade of phosphorylation and ubiquitination PTMs. Intermolecular autophosphorylation at Ser318 and trans-phosphorylation mediated by BIK1 form the core phosphorylation modification network of CPK28. Research indicates that these phosphorylation events determine the interaction efficiency between CPK28 and the E3 ubiquitin ligase ATL31/6. Phosphorylation-defective mutants (e.g., Ser318Ala or mutations at BIK1 sites) weaken the CPK28-ATL31 binding capacity, leading to reduced ubiquitination levels and delayed proteasomal degradation. Concurrently, CPK28 forms disulfide bonds via intermolecular interactions, and its assembly status is inversely regulated by the extent of CPK28 phosphorylation. This phosphorylation-dependent ubiquitination degradation mechanism exemplifies antagonistic crosstalk between PTMs (Liu et al., 2023). NO regulates the assembly of the SCF^TIR1/AFB^ ubiquitin ligase complex through redox modifications, thereby influencing auxin signaling. The Cys37 and Cys118 residues of the ASK1 protein serve as sites for redox modifications, undergoing S-nitrosylation and S-glutathionylation. NO-mediated S-glutathionylation directly promotes the binding of ASK1 to CUL1 and TIR1/AFB2. This enhanced binding is essential for the correct assembly of the SCF^TIR1/AFB^ ubiquitin ligase complex (Iglesias et al., 2018). Stomatal regulation is controlled by the MAPK cascade and NO, which exert both negative and positive control. In *Arabidopsis*, NO-mediated stomatal development depends on MPK3 and MPK6. S-nitrosylation at Cys-201 of MPK6 by NO inhibits its phosphorylation, thereby promoting stomatal development (Wang et al., 2025).

In a previous study, the casein kinase II α subunit (CPCK2) was reported to function as a negative regulator of innate immunity in *Arabidopsis*. Researchers found that CPCK2 interacted with the chloroplast-localized carbonic anhydrase (CA) and SA-binding protein 3 (SABP3), which is essential for CPCK2-mediated immunity. CPCK2 phosphorylates SABP3, thereby promoting the S-nitrosylation of this enzyme. Previous findings demonstrate that CPCK2 acts as a negative regulator of SA accumulation and associated immunity through the phosphorylation-dependent promotion of SABP3 S-nitrosylation (Rui et al., 2024).

Plants dynamically regulate protein functions through PTM crosstalk networks, in which OxiPTMs serve as a central hub integrating environmental signals and developmental cues to enable precise physiological responses.

### 4.2 CCM remodeling mediated by PTMs

In plants, PTMs remodel CCM by regulating enzyme activity. Phosphorylation, acetylation, and redox regulatory sites of multiple CCM enzymes have been reported (Bykova et al., 2003; König et al., 2014). Among these, the GAPDH family exhibits high redox sensitivity. Under oxidative stress, phosphorylated GAPDH catalyzes the dephosphorylation of its BPGA substrate (1,3-bisphosphoglycerate). In addition, the plant cytosol contains non-phosphorylated GAPDH (GAPN), which directly oxidizes glyceraldehyde-3-phosphate (G3P) to 3-phosphoglycerate, bypassing the reaction catalyzed by cytosolic glyceraldehyde-3-phosphate dehydrogenase (GAPC). Plant cytosolic NAD-dependent GAPC undergoes redox-dependent conformational changes that promote the transfer of substrate G3P to mitochondria, driving ATP production. Plant GAPN has been expressed in G6PDH-deficient yeast, successfully compensating for the lack of G6PDH, maintaining the NADPH/NADP^+^ ratio, and enhancing yeast resistance to oxidative stress. This demonstrates that GAPN functionally replaces G6PDH to generate NADPH, reducing reliance on conventional PPP under stress conditions (Schneider et al., 2018). This kind of metabolic flexibility enables plants to maintain the NADPH pool under oxidative stress. As a phosphorylated NAD-specific GAPDH, GapC1 regulates its activity through redox modifications at Cys149, linking cellular metabolism to the oxidative stress response. The thiolate group (-S^−^) of Cys149 is readily oxidized by H_2_O_2_, first forming an unstable sulfenate intermediate (-SO^−^), which is then irreversibly converted into –SO_2_H and –SO_3_H, resulting in the loss of enzymatic activity. However, GSH can react with the sulfenate intermediate (-SO^−^) at Cys149 to form a mixed disulfide bond (–SS–), preventing irreversible oxidation (Bedhomme et al., 2012) (**Table 1**). In plant mitochondria, the targeted phosphatases PP_2_c6_3_ and Sal2 are involved in the dephosphorylation of the pyruvate dehydrogenase complex (PDC). PP_2_c6_3_ directly regulates PDC activity through dephosphorylation, influencing the metabolic flux of the TCA cycle. In contrast, the phosphatase Sal2 indirectly affects enzymes in the TCA cycle, demonstrating that the PTMs of enzymes in CCM modulate the metabolic flux of this pathway. Changes in TCA cycle intermediates have been measured using isotope labeling, and the results are consistent with altered PDC activity (Zhang et al., 2021). In the PP_2_c6_3_ and *Sal2* mutants, the states of enzymes outside the TCA cycle are also altered.

**Table 1:** PTM types and functions of CCM enzymes.

CCM enzyme	PTM type	Function	Reversibility	Reference
GapC1	Sulfenylation	Oxidative inactivation of enzyme active sites	Reversible	Bedhomme et al., 2012
GapC1	S-glutathionylation	Protects enzymes from permanent inactivation	Reversible	Bedhomme et al., 2012
GapC1	Sulfinylation	Loss of enzyme activity	Irreversible	Bedhomme et al., 2012
GapC1	Sulfonylation	Loss of enzyme activity	Irreversible	Bedhomme et al., 2012
G6PD	Persulfidation	Alters spatial structure	Reversible	Wang et al., 2023
MDH	Disulfide bond formation	Inhibits enzyme activity	Reversible	Huang et al., 2018
MDH	Oxidative modifications	Restores enzyme activity	Reversible	Huang et al., 2018
GAPC	Enhances the endogenous antioxidant defense ability	Alters the compartment localization of the enzyme in question	Reversible	Schneider et al., 2018
ENO2	Sulfinylation	Promotes oligomer formation and nuclear translocation	Reversible	(Liu et al., 2022a)

The flexibility of CCM mediated by PTMs is also reflected in antioxidant defense. In this process, the flux of the PPP pathway is primarily regulated by changes in the expression or enzymatic activity of G6PD. Hydrogen sulfide (H_2_S) enhances the antioxidant stress capacity by regulating G6PD in the plant cytosol through persulfidation modifications. Under salt stress, persulfidation modifications occur at Cys159 in *Arabidopsis* G6PD6 and Cys155 in tomato G6PDC, which alter the spatial structure of G6PD, specifically shortening the distance between lysine residues (K491–K475) in the NADP+-binding domain. They consequently enhance G6PD oligomerization and substrate affinity, protect G6PD from oxidative damage, and maintain enzymatic activity (Wang et al., 2023) (**Table 1**). Research on the PTMs of G6PD in plants is limited, but studies have been reported in other organisms. Histone PTMs serve as critical epigenetic mechanisms for regulating the transcriptional activity of G6PD. A study in humans revealed that acetylation is involved in the transcriptional regulation of G6PD. Inhibition of histone deacetylases significantly increases the acetylation levels of histones H3/H4 in the promoter region of G6PD, facilitating recruitment of the transcription factor Sp1 to the G6PD promoter. This enhances recruitment of RNA polymerase II and assembly of the transcription initiation complex, driving the upregulation of G6PD gene expression. This mechanism successfully restores enzymatic activity in cells of G6PD-deficient patients (Makarona et al., 2014). H_2_O_2_ has also been implicated in G6PD activation and overexpression, although the underlying mechanism remains unclear (Chettimada et al., 2015).

As previously mentioned, ROS exhibit compartment-specific distribution in plant cells, and enzymes in different compartments have varying sensitivities to oxidative modifications. In the TCA cycle, Cys and methionine (Met) residues of malate dehydrogenase (MDH) are prone to oxidation. H_2_O_2_ inhibits MDH activity by oxidizing its Cys330 and Met residues. Cytosolic Trx specifically reduces the intermolecular disulfide bond (–SS–) formed at Cys330, restoring enzyme activity and protecting it from overoxidation. This regulatory mechanism is compartment specific, with cytosolic and chloroplast plNAD-MDH being sensitive to H_2_O_2_ and mitochondria mMDH lacking this property. mMDH relies on the CAT2 and thioredoxin reductase systems to maintain a dynamic redox balance. This compartmentalized regulation enables plants to rapidly modulate MDH activity during oxidative stress, thereby adapting to environmental challenges (Huang et al., 2018) (**Table 1**). In addition, OxiPTMs alter the compartmental localization of related enzymes. GAPC, which dependent on oxidative modifications of its catalytic Cys residues, exhibits a significant increase in nuclear localization, suggesting that OxiPTMs mediate enzyme relocalization, which is linked to metabolic compartmentalization (Schneider et al., 2018) (**Table 1**). Phosphoenolpyruvate, the direct precursor of pyruvate in the EMP pathway, is generated by the conversion of 2-phosphoglycerate catalyzed by enolase (ENO). This process represents a critical step prior to the entry of the EMP pathway into the TCA cycle. Under low-temperature stress, H_2_O_2_ induced in plants mediates sulfinylation at Cys408 of ENO2, promoting oligomer formation and nuclear translocation, enhancing its binding and activation of the cold stress key regulatory gene CBF1 (C-repeat-binding factor 1) (Liu et al., 2022a) (**Table 1**).

Numerous PTM sites have been identified in plant proteins, but the mechanisms of PTMs in CCM enzymes are only known for a few. As a central hub in stress responses, ROS coordinate CCM remodeling by either directly mediating or indirectly influencing PTMs (Bright et al., 2006; Corpas et al., 2022; Zhou et al., 2023). However, research on the crosstalk mechanisms between ROS and PTMs in plants remains insufficient, highlighting the need for deeper exploration of this vast metabolic network.

## 5 Conclusion and prospects

Given the complex and dynamic environments that plants face, there are still unexplored areas regarding how CCM supports survival mechanisms under simultaneous exposure to multiple environmental factors. Due to the specificity of ROS, how ROS-mediated PTMs differ temporally between early and late stress stages and how ROS-mediated PTMs in distinct cellular compartments operate spatially independently yet interactively remain unclear. As one of the key mechanisms by which ROS influences CCM, how varying levels of ROS-mediated PTMs further modulate CCM is still unknown. In addition, whether CCM feedback regulates ROS homeostasis through PTMs should be verified. Current approaches rely largely on genetic or traditional protein interaction techniques to identify enzymatic changes in CCM. By targeting the identified PTM sites in CCM enzymes, future research should design abiotic stress-adapted ROS-PTM-CCM networks for crops through (1) determining the spatiotemporal dynamics of ROS-PTM-CCM under combined stresses; (2) engineering PTM sites in CCM enzymes to support the development of stress-resilient crops; and (3) implementing the targeted chemical regulation of CCM enzymes.
